# Renal Pseudoaneurysms after Flexible Ureteroscopy and Holmium Laser Lithotripsy: A Case Report

**DOI:** 10.3389/fsurg.2022.896548

**Published:** 2022-05-12

**Authors:** Xin-xi Deng, Wensheng Zhang, Delai Fu, Bin Fu

**Affiliations:** ^1^ Department of Urology, Jiu Jiang NO.1 People’s Hospital, Jiujiang, China; ^2^ Department of Urology, The First Affiliated Hospital of Nanchang University; Jiangxi Institute of Urology, Nanchang, China; ^3^ Department of Urology, The Second Affiliated Hospital of Xi’an Jiaotong University, Xi’an, China

**Keywords:** flexible ureteroscopy (FURS), holmium laser, pseudoaneurysm (PA), renal calculi, vascular embolization

## Abstract

**Background:**

Flexible ureteroscopy (FURS) and holmium laser lithotripsy is considered one of the most minimally invasive and safe surgical methods for the treatment of renal calculi. Renal pseudoaneurysm is a rare complication after FURS holmium laser lithotripsy. We report a case of renal pseudoaneurysm after FURS and holmium laser lithotripsy and review the relevant literature to analyze the possible etiology and summarize the treatment.

**Case presentation:**

A 29-year-old male with a 2-year history of diabetes was admitted to the hospital because of right back pain for 5 days. A doppler ultrasound demonstrated bilateral renal calculi with bilateral mild hydronephrosis. The patient underwent one-stage right FURS and holmium laser lithotripsy and bilateral ureteral stent implantation. The urine was clear on the second day after the operation, and he was discharged from the hospital. Due to severe gross hematuria, he had to be hospitalized 28 days after the operation. A CT scan showed multiple blood clots in the right renal pelvis and bladder. An emergency blood transfusion and removal of the bladder blood clots and bilateral double J tubes were performed. His urine was clear for one week, and he was discharged from the hospital. He was hospitalized again 47 days after the operation because of fever and hematuria. A CT scan demonstrated blood clots in the bladder and right renal pelvis. Angiography showed a pseudoaneurysm in a small branch of the right renal artery, and hematuria stopped after selective renal artery embolization with microcoils.

**Conclusion:**

FURS and holmium laser lithotripsy is safe, but we should be aware of the possibility of renal artery pseudoaneurysms (RAP). Through careful operation during the surgery, avoiding kidney injury, reducing intrarenal pressure and controlling the time of operation may prevent the occurrence of this complication. Vascular embolization is the first choice for the treatment of pseudoaneurysms.

## Introduction

FURS and holmium laser lithotripsy is widely used for the treatment of renal calculi with minimal trauma and rapid recovery. The treatment is suitable for renal stones less than 2 cm and residual stones after shock wave lithotripsy (SWL) or percutaneous nephrolithotripsy (PCNL). Compared with FURS lithotripsy, PCNL has some disadvantages, such as a high risk of bleeding and slow recovery after the operation (1). Therefore, there are an increasing number of reports about FURS lithotripsy in the treatment of renal calculi larger than 2 cm (2). However, RAP caused by FURS lithotripsy is rare. We report a case of pseudoaneurysm after FURS and holmium laser lithotripsy, and the related literature was reviewed by PubMed and other databases (3–7) (Supplementary Table S1). The clinical characteristics of the patient were analyzed, and the possible etiology and exact treatment of the pseudoaneurysm have been summarized.

## Case Presentation

The patient was a 29-year-old male with a 2-year history of diabetes. He was admitted to the hospital because of right back pain for 5 days. A doppler ultrasound showed bilateral renal calculi and bilateral mild hydronephrosis. The size of the right kidney stone was approximately 2.5*2.2 cm, and the size of the left kidney stone was approximately 2.2*2.3 cm. A CT scan showed double renal calculi with mild hydronephrosis, double renal pelvis and upper ureteral inflammation (Supplementary Figure S1). Routine examinations were basically normal before the operation. The patient underwent one-stage right FURS and holmium laser lithotripsy and double ureteral stent implantation under general anesthesia. During the operation, an ultraslippery hydrophilic guide wire (0.35 mm, Cook, USA) and a ureteral access sheath (inner diameter: 12 Fr, length: 46 cm, Laikai Medical, China) were inserted into the right ureteropelvic junction. Using hand-pump irrigation, a flexible ureteroscope (8.5 Fr, Karl Store, Germany) showed a yellowish-brown stone in the right renal pelvis approximately 2.0*2.5 cm in size. The holmium laser energy was adjusted to 0.8 J, and the frequency was 25 Hz. The stone was crushed into powder, and the double J tube was indwelled. The operation time was approximately 60 min, and no active bleeding was observed during the operation. The patient’s urine was clear, and he was discharged on the second day after the operation.

The patient came to the emergency department of our hospital 28 days after the operation due to severe gross hematuria with urinary retention. A CT scan indicated multiple stones in the bilateral kidneys with mild hydronephrosis, and massive blood clots were considered in the right renal pelvis and bladder (Supplementary Figures S2A,B). The hemoglobin level decreased sharply from 165 to 68 g/L. First, 8 units of red blood cells were transfused to maintain the patient’s vital signs, and the bladder blood clots and bilateral double-J tubes were removed in emergency surgery. A large amount of blood clots in the bladder and intermittent bloody urine in the bilateral ureteral opening could be seen. After the operation, the bladder was continuously irrigated with three cava balloon urinary catheters, the urine was clear, and the hemoglobin level was not further decreased. This patient refused angiography and was discharged after the operation for one week.

**Figure 1:**
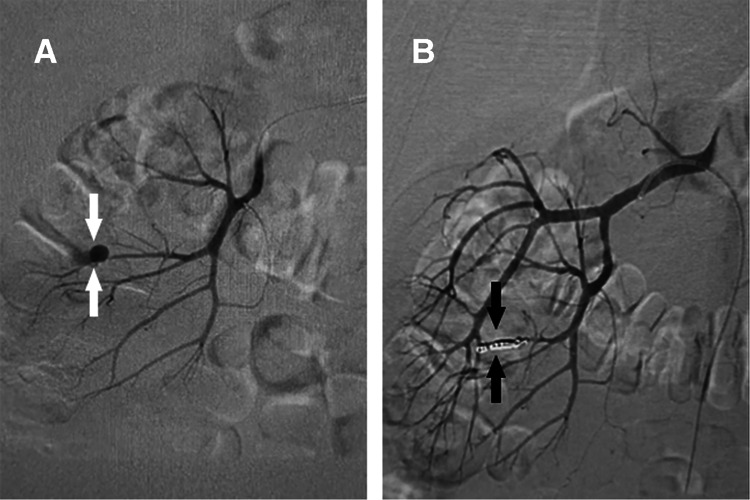
(**A**) An angiogram showing a right interlobar RAP (white arrows). (**B**) Microcoils embolizing the pseudoaneurysm in the right kidney (dark arrows).

He was hospitalized again 47 days after the operation due to complaints of fever and hematuria. A CT scan demonstrated a large amount of blood clots in the right renal pelvis and bladder and right perirenal fascia thickening (Supplementary Figures S2C,D). Considering the recurrence of active bleeding, angiography showed a pseudoaneurysm of a small branch of the right renal artery (Figure 1). After selective arterial embolization with microcoils, the patient recovered and was discharged one week later. No obvious abnormality was found during the 2-month follow-up, and the patient did not consider the operation for a left kidney stone for the time being.

## Discussion

RAP is a pulsatile hematoma formed by blood outflow from the rupture of the renal artery wall and is wrapped by perirenal soft tissue (5). The wall of an RAP tumor is not a real blood vessel wall but a cystic wall formed by the organization of tissue around the hematoma. With the progressive increase in bleeding, the pulling force on the cyst wall gradually increases, resulting in the sudden rupture of the cyst wall, causing massive bleeding, and even endangering the lives of patients. The occurrence of RAP is often caused by abdominal trauma or kidney injury after renal surgery (8). For example, after PCNL, the incidence of intrarenal pseudoaneurysm or arteriovenous fistula is approximately 0.6%–1.0% (9). However, this complication is extremely rare after FURS and holmium laser lithotripsy. In total, we obtained 6 cases of pseudoaneurysm after FURS, of which 5 cases were retrieved by PubMed and 1 case was reported by us. There were 2 cases of electrohydraulic lithotripsy and 4 cases of holmium laser lithotripsy. The ages of the patients ranged from 29 to 79 years, including 5 males and 1 female. Four patients had diabetes, high blood pressure or cardiovascular diseases, and 2 patients had no reported underlying diseases. No obvious active bleeding was found in any patient during the operation, and the operations were completed successfully. All patients showed delayed gross hematuria, which was cured by renal angiography and vascular embolization. However, the specific causes of its formation are still unclear, and it is considered that the possible causes include intraoperative thermal injury, mechanical injury, irrigation pressure, operation time and some underlying diseases affecting renal vessels, resulting in the formation of postoperative pseudoaneurysms.

RAP often manifests as delayed hematuria in the clinical setting. After vascular injury during the operation, there was no obvious bleeding in the short term because the compression of the surrounding tissue covers the injury of diseased blood vessels. When blood pressure rises, the wall of the damaged artery will rupture and bleed (10). In all cases, the bleeding time ranged from 6 days to 46 days. It was previously reported that this is related to the method of lithotripsy (11). For example, electrohydraulic hydroelectricity is more likely to damage the kidney than the holmium laser during lithotripsy, but with the widespread use of holmium laser, 4 cases of pseudoaneurysm after holmium laser lithotripsy have been reported. Both lithotripsy methods may lead to renal vascular injury and the formation of pseudoaneurysms. The thermal damage caused by the holmium laser energy should not be ignored. Tiplitsky et al. (12) proposed that laser thermal injury can avoid direct renal injury by maintaining a distance between the optical fiber and the mucous membrane of at least 0.5 mm, while the use of low energy levels can minimize renal vessel damage (4).

Second, mechanical damage to the kidney during the use of guide wires has also attracted more attention. If an inappropriate guide wire with hard or poor flexibility is chosen, the wire may damage the kidney. However, with the development of technology and materials, super-slippery guide wires have appeared, and the possibility of kidney damage caused by guide wires is lessened (5); however, they cannot be completely avoided. This may be related to the improper use of the guide wire during the operation or the pathological changes of the kidney itself. In our case, a super-slippery hydrophilic guide wire was selected, and there was no renal parenchyma injury during the operation. The position of the double-J tube was normal after the operation, and the possibility of a guide wire injury was not considered. Some related diseases lead to pathological changes in the kidney, making it easier for guide wires to penetrate the renal parenchyma, especially in patients with long-term vascular diseases such as diabetes, high blood pressure and coronary heart disease. In addition, due to long-term severe hydronephrosis, the renal cortex is thinner and prone to kidney damage.

At the same time, the intrarenal perfusion pressure and operation time are also issues that should be considered during FURS. These two factors are very important for septic shock, but they may also be related to the formation of postoperative pseudoaneurysms. As we all know, the normal physiologic intrarenal pressure is approximately 10 mmHg. When a hand-pump rather than an intelligent pressure pump is used during the operation, it is easy to increase the intrarenal pressure and lead to rupture of the renal parenchymal or fornix and renal hemorrhage (13). For pathological renal damage, renal hemorrhage may occur even when the intrarenal pressure is normal. In addition, if the ureteroscope sheath is not inserted or if there is a size mismatch between the ureteroscope sheath and the flexible ureteroscope, it can still lead to an increase in intrarenal perfusion pressure (14). This leads to renal dilatation and parenchyma thinning during the operation, which aggravates the damage to blood vessels. For example, long-term operation (15) will lead to renal ischemia, affect the elasticity of the blood vessel wall, and aggravate vascular injuries. To date, 6 cases have been reported, of which 4 cases were not given close attention to the operation time and pumping method. One patient was operated on for more than 90 min, and the water pressure was controlled within 80 cm of H_2_O. In another case, the operation time was less than 90 min, and the water pressure was controlled by artificial water injection. Therefore, the risk may be reduced by controlling the appropriate intrarenal pressure and operation time during the operation.

Vascular embolization is currently considered to be the first-line treatment of renal pseudoaneurysms in patients with hemodynamic stability because it is efficient and safe and has little effect on renal function (6). In all 6 cases reported, the patients were discharged from the hospital after vascular embolization and recovered well after the operation. Therefore, vascular embolization should be the first choice for the treatment of intrarenal artery pseudoaneurysms.

## Conclusion

In summary, although the occurrence of pseudoaneurysms after FURS and holmium laser lithotripsy is very rare, it should not be ignored. For example, patients with obvious hematuria, severe back pain or hemodynamic instability, pseudoaneurysms need to be highly suspected. During the operation, a clear field of vision, as far as possible, should be maintained, laser damage to the kidney should be avoided, the laser energy should be reduced, mechanical injuries caused by the guide wire should be avoided, and the operation time and intrarenal pressure should be controlled, especially in patients with potential diseases that affect the integrity of renal vessels. Once pseudoaneurysms happen, renal arteriography and vascular embolization should actively be performed.

## Data Availability

The original contributions presented in the study are included in the article/Supplementary Material, further inquiries can be directed to the corresponding author/s.
