# Supplementary material for: Renal Pseudoaneurysms after Flexible Ureteroscopy and Holmium Laser Lithotripsy: A Case Report
Source: Front Surg. 2022 May 12;9:896548. doi: 10.3389/fsurg.2022.896548 (PMC9406514; doi:10.3389/fsurg.2022.896548)

Figure Captions

Supplementary Figure 1. (A) A preoperative computed tomography (CT) scan revealed a double renal stone with mild hydronephrosis. (B) The postoperative kidney ureter bladder (KUB) showed a normal position of the double-J tubes and multiple fragmentary stones in the right kidney.

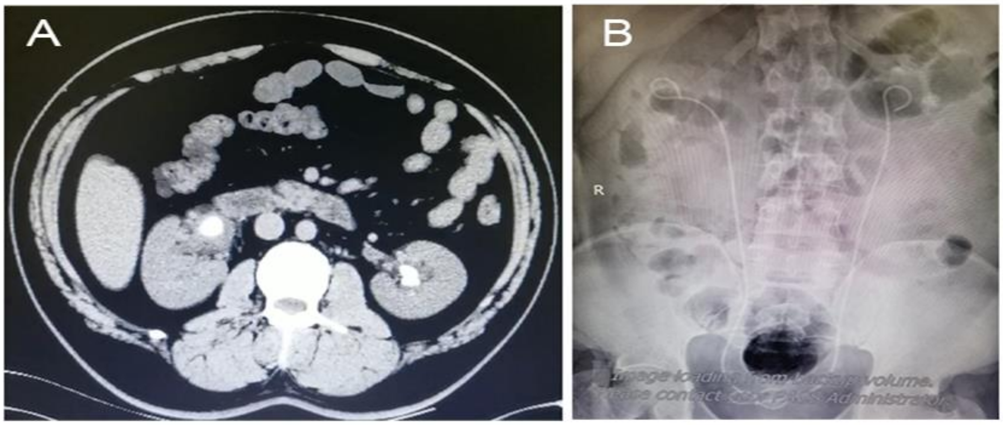

Supplementary Figure 2. A large amount of blood clots was observed in the right renal pelvis and bladder at 28 days (A, B) and 47 days (C, D) after the operation.

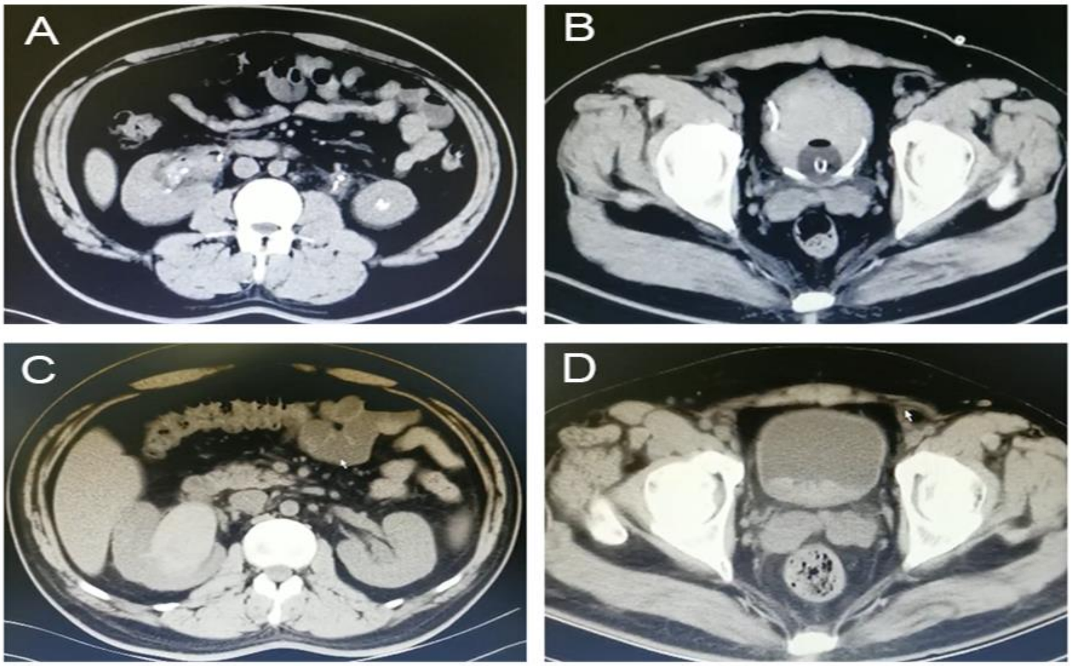

Supplement: Supplementary file 1 [file Data_Sheet_1_v1.pdf]
