# Supplementary material for: Renal Pseudoaneurysms after Flexible Ureteroscopy and Holmium Laser Lithotripsy: A Case Report
Source: Front Surg. 2022 May 12;9:896548. doi: 10.3389/fsurg.2022.896548 (PMC9406514; doi:10.3389/fsurg.2022.896548)
Supplement: Supplementary file 2 [file Table_1_v1.docx]

**Supplementary Table 1.** Clinical data of the 6 patients with renal pseudoaneurysm after FURS lithotripsy.

| **Author**  **(years)** | **Gender** | **Age** | **History disease** | **stone burden**  **(cm)** | **Pump water method** | **Lithotripsy method** | **Laser energy** | **Operation time**  **(min)** | **Clinical feature** | **Embolization time**  **(days)** |
| --- | --- | --- | --- | --- | --- | --- | --- | --- | --- | --- |
| Rudnick DM et al.  (1998) | Male | 49 | NR | NR | NR | EHL | NR | NR | Persistent Hematuria | 21 |
| Aston W et al.  (2004) | Male | 29 | NR | NR | NR | EHL | NR | NR | Persistent Hematuria, Pain | 46 |
| Durner L et al.  (2017) | Male | 56 | CHD | 1 | NR | HLL | NR | NR | Persistent Hematuria | 14 |
| Jubber I et al.  (2018) | Female | 68 | NSTEMI | 1.1 | NR | HLL | NR | NR | Vomiting,  Abdominal, Pain, Fever | 21 |
| Bashar A et al.  (2019) | Male | 79 | HBP, HD, DM, CKD | 3.2 | 80cmH2O | HLL | 1.0-1.2J  8-12Hz | 125 min (Left:65 min, Right:60min) | Hematuria, Pain | 6 |
| Xinxi Deng et al.  (this case) | Male | 29 | DM | 2.5 | Hand-pump irrigation | HLL | 0.8J  25Hz | 60min | Hematuria, Pain, Fever | 47 |

**Notes:** NR, Not Reported; NSTEMI, Non-ST elevation myocardial infarction; HBP, Hypertension; CHD, Congenital valvular heart disease; DM, Diabetes mellitus; CKD, Chronic kidney disease; EHL, Electrohydraulic lithotripsy; HLL, Holmium laser lithotripsy.
